# New Onset On-Medication Freezing of Gait After STN-DBS in Parkinson's Disease

**DOI:** 10.3389/fneur.2019.00659

**Published:** 2019-06-19

**Authors:** Shanshan Mei, Jiping Li, Erik H. Middlebrooks, Leonardo Almeida, Wei Hu, Yuqing Zhang, Adolfo Ramirez-Zamora, Piu Chan

**Affiliations:** ^1^Department of Neurology, Xuanwu Hospital of Capital Medical University, Beijing, China; ^2^Department of Functional Neurosurgery, Xuanwu Hospital of Capital Medical University, Beijing, China; ^3^Department of Radiology, Mayo Clinic, Jacksonville, FL, United States; ^4^Department of Neurologic Surgery, Mayo Clinic, Jacksonville, FL, United States; ^5^Department of Neurology, Fixel Institute for Neurological Diseases, University of Florida, Gainesville, FL, United States

**Keywords:** deep brain stimulation, subthalamic nucleus, on-state, freezing of gait, fiber tracking, prefrontal cortex, cerebellum

## Abstract

Freezing of gait (FoG) is commonly observed in advanced Parkinson's disease (PD) and it is associated with reduced mobility, recurrent falls, injuries, and loss of independence. This phenomenon typically occurs as the effect of dopaminergic medications wears off (“off” FoG) but on rare occasions, it can also be observed during peak medication effect (“on” FoG). In this report, we present the case of a 65-year-old female with a 13-year history of akinetic-rigid idiopathic PD who developed recurrent episodes of “on” FoG after bilateral subthalamic nucleus deep brain stimulation (STN-DBS). She underwent STN-DBS for management of motor fluctuations, which resulted in a marked improvement in her motor symptoms. Within the next 6 months and after several programming sessions, the patient reported “on” FoG occurring regularly 1 h after taking levodopa and lasting a few hours. Accordingly, a repeated levodopa challenge showed that FoG resolved with either levodopa administration or STN stimulation alone, but the combination of both therapies induced recurrence of FoG in our patient. Subsequent management was complex requiring adjustments in levodopa dose and formulation along with advanced DBS programming.

## Introduction

Freezing of gait (FoG) is a common and disabling symptom in Parkinson's disease (PD) in which patients are unable to initiate or continue locomotion (1). It is defined as “brief, episodic absence or marked reduction of forward progression of the feet despite the intention to walk” (2). It appears at the initiation of gait or during locomotion resulting in the inability to lift the feet from the floor and trembling of the legs (3). This complex motor phenomenon can occur at any point in the course of the disease, but it is more commonly observed in advanced PD (1, 4). FoG is frequently observed at the end of dose or “off” medication state, as the dopaminergic medication effect is wearing off. Less frequently, FoG might occur during peak medication effect or “on” medication state (5–7).

Subthalamic nucleus deep brain stimulation (STN-DBS) is a well-established therapy for motor fluctuations and PD symptoms including akinesia, rigidity, and tremor. However, the efficacy of STN-DBS on axial symptoms such as FoG, postural instability, and gait impairment has been inconsistent with conflicting reports (8, 9). Although STN-DBS frequently improves levodopa-responsive symptoms including FoG (10), there are reported cases of persistent or even worsening FoG after DBS surgery (8, 11). Additionally, acute FoG can be induced by STN-DBS, possibly related to the disruption of afferent fibers from the pedunculopontine nucleus (PPN) to STN (12).

There is limited information regarding the occurrence of FoG due to the simultaneous use of levodopa and STN-DBS. We describe a case in which levodopa and STN-stimulation independently resolved “off” FoG, however, the combined effect of both therapies consistently induced “on” FoG that required further medication adjustments and advanced DBS programming. Lastly, we also discuss the potential neuroanatomic mechanisms responsible for this unique interaction.

## Case Presentation

A 65-year-old female with a 13-year history of akinetic-rigid idiopathic PD presented with worsening parkinsonism, motor fluctuations, and “off” FoG. Early in the disease course, treatment with levodopa and selegiline resulted in excellent therapeutic benefit. Eight years after the diagnosis, unpredictable motor fluctuations, “off” dystonia, lower extremities peak-dose dyskinesia, and “off” FoG became increasingly difficult to manage with medical therapy. In preparation for DBS, after an overnight withdrawal of dopaminergic medications, a levodopa challenge test with 400 mg levodopa (13) showed a 72% improvement in her total Movement Disorder Society Unified Parkinson's Disease Rating Scale (MDS-UPDRS; 14) motor score from (55 points in the off-medication (off-med) state to 15 points in the on-medication (on-med) state). Importantly, the item 3.11 of MDS-UPDRS (FoG score) improved from 4 to 0.

**Figure 1:**
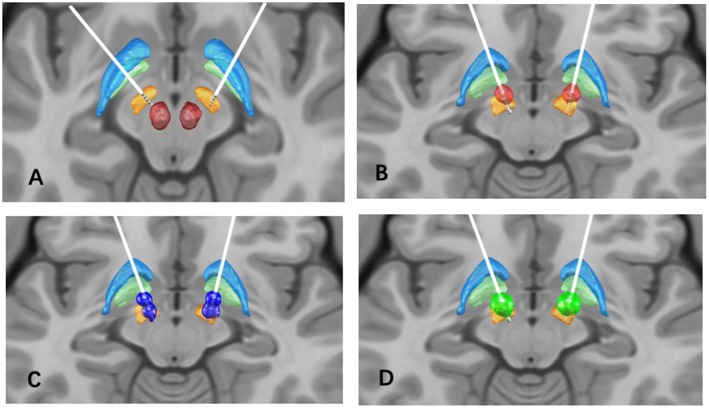
**(A)** Bilateral STN lead location, the STN (orange), globus pallidus internus (light green), globus pallidus externus (light blue), and red nucleus (red) are shown for reference; **(B)** Volume of tissue activated (VTA) in the initial programming setting (red), L-STN (C+;2-), R-STN (C+;10-), 60 us pulse width, 130 Hz frequency, and 3.0 V; **(C)** VTA in the second programming setting (dark blue): L-STN (C+;1-;3-), R-STN (C+;9-;11-), 90 us pulse width, 130 Hz frequency, and 2.0V; **(D)** VTA in the final programming setting (green): L-STN (C+;2-), R-STN (C+;10-), 90 us pulse width, 60 Hz frequency, and 4.5 V.

After a multidisciplinary assessment, the patient underwent bilateral placement of STN-DBS leads (3389, Medtronic, Minneapolis, Minnesota USA; Figure 1A). This provided an excellent clinical response with adequate thresholds for side effects, corticospinal side effects around 3–4V in all contacts. Dyskinesia of the left foot was noted with the programming of left contacts 0 and 1, and generalized dyskinesia with right contact 9. Three months after surgery, motor symptoms continued to be improved with stable stimulation parameters: amplitude 3.0 V, pulse width 60 μs, frequency 130 Hz in monopolar mode bilaterally with left STN (C+;2-) and right STN (C+;10-) (Figure 1B). The motor MDS-UPDRS score decreased to 37 points in the off-medication and on-stimulation (off-med/on-stim) condition (33% improvement). Subsequently, within 6 months after surgery, the patient reported a consistent appearance of “on” FoG, after taking medications and lasting several hours. It should be noted that preoperatively, the patient had only “off” FoG. Multiple programming sessions and attempts of double monopolar stimulation using different amplitudes (Figure 1C) provided transient benefit in her FoG.

**Figure 2:**
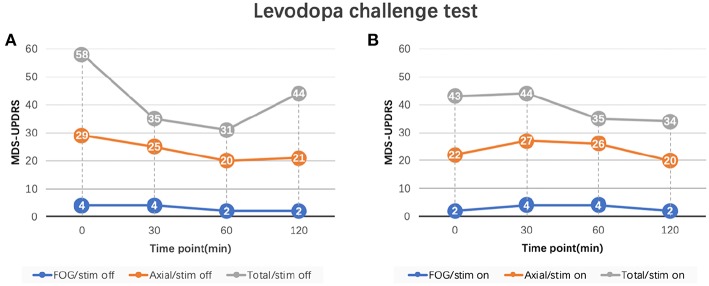
MDS-UPDRS sub-scores of levodopa challenge test. **(A)** FoG and axial scores of levodopa challenge test in the off-stimulation state. **(B)** FoG and axial scores of levodopa challenge test in the on-stimulation state. FoG score: MDS-UPDRS item 3.11; Axial score: the sum of MDS-UPDRS item 3.1 (speech), 3.2 (facial expression), 3.3 (neck), 3.9 (arising from chair), 3.10 (gait), 3.11 (FoG), 3.12 (posture stability), 3.13 (posture), and 3.14 (body bradykinesia); Total score: MDS-UPDRS part III total score. Stimulation parameters: L-STN (C+;1-;3-), R-STN (C+;9-;11-), 90 us pulse width, 130 Hz frequency, and 2.0 V. MDS-UPDRS, Movement Disorder Society Sponsored Revision of the Unified Parkinson's Disease Rating Scale; FoG, freezing of gait.

In order to determine whether FoG was caused by neurostimulation or dopaminergic medications, the levodopa challenge test with a same dosage as pre-operation was repeated in the on-stimulation (on-stim) and off-stimulation (off-stim) conditions. Patient's MDS-UPDRS FoG score was 2 in the off-med/on-stim state and 4 in the off-med/off-stim state. Once stimulation was turned off, the patient developed a recurrence of FoG which gradually improved after levodopa (FoG score improved from 4 to 1), along with other axial symptoms (Figure 2). The FoG was reproducibly improved either by levodopa (on-med/off stim) or neurostimulation (off-med/on-stim). In the on-med/on-stim state, the patient developed persistent, severe FoG and gait difficulties for several hours after levodopa intake [FoG score increased from 2 to 4 with the axial score from 22 to 27] (Figure 2).

We determined that the summative effect of electrical stimulation and dopaminergic stimulation induced “on” FoG, similar to previously reported FoG induced by supratherapeutic levodopa doses (7). Initial management strategies included reduction of levodopa dose and the use of extended-release preparations with partial improvement in FoG. We then adjusted the stimulation and applied low-frequency settings as follows: Left STN (C+;2-), Right STN (C+;10-), amplitude 4.5 V, pulse width 90 μs, and frequency 60 Hz (Figure 1D). At 1-year post-operation follow-up, her FoG scores were significantly improved in both the medication on and off states (FoG score improved from 4 to 1).

In order to localize the specific anatomical electrode positioning and determined the volume of tissue activated (VTA), patient's pre-operative T1-weighted magnetization prepared rapid gradient echo (MP-RAGE) images were co-registered to her post-operative high-resolution CT scan prior to normalization to the Montreal Neurological Institute (MNI) template space using the Lead-DBS software package (14). The STN was localized by means of the DBS Intrinsic Template Atlas (DISTAL) (15). Lead-DBS was utilized to simulate VTAs for all presented DBS programming settings using a tissue-specific conductivity model (14), which were used as seed volumes for deterministic tractography in DSI Studio (http://dsi-studio.labsolver.org/). A group-averaged diffusion dataset concatenated from 1,065 normative datasets in the Human Connectome Project database (https://db.humanconnectome.org) was utilized for fiber tracking. A total of 5,000 tracts were generated from each VTA and fiber counts were measured to target regions in the Automated Anatomical Labeling (AAL) atlas (16) and the cerebellar spatially unbiased atlas template (SUIT) (17).

**Figure 3:**
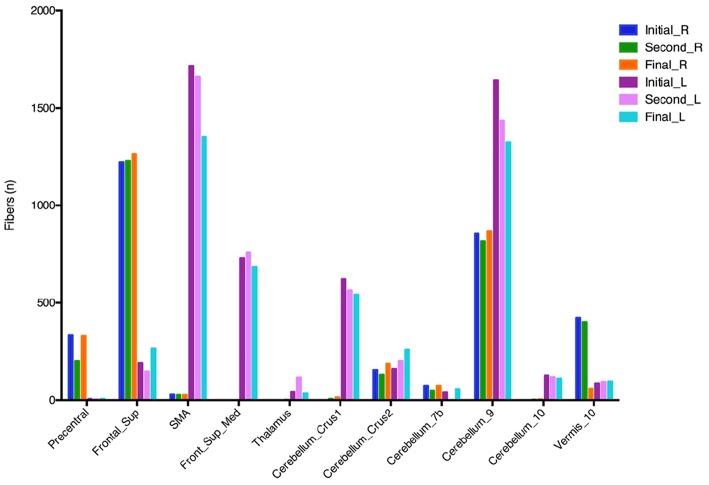
Fiber tracking from different VTAs of three programming settings. VTA, volumes of tissue activated; Frontal Sup, superior frontal gyrus; Frontal_Sup_Med, medial frontal gyrus; SMA, supplementary motor area. The initial, second, and final programming settings are referred to Figure 1.

The fiber tracking generated from the different VTAs of the three programming settings revealed overall greater connectivity between the left VTAs to the medial frontal gyrus, supplementary motor area (SMA), and multiple regions of the cerebellum when compared to the right VTA. Notably, there was a decrease in the connectivity of the left VTA and these regions during the improvement of “on” FoG (Figure 3). According to these findings, we hypothesize that the changes in the neuro-connectivity affecting the prefrontal cortex (PFC) and cerebellum in the setting of the combination of neurostimulation and levodopa lead to the occurrence of “on” FoG.

## Discussion

We present a case of unanticipated, acute, reproducible “on” FoG caused by the combined effect of levodopa and STN-DBS. FoG might occur following STN-DBS or be related to disease progression (4), but to the best of our knowledge, there are no reports of “on” FoG caused by the combination of medications and DBS. Most commonly, “on” FoG is observed as a consequence of disease progression, limited management of parkinsonism and suboptimal placed leads (18). Several cases of acute “on” FoG after DBS have been reported, which may be related to the DBS electrode passing through the fields of Forel and subsequently damaging the afferent fibers from the PPN to STN (12).

There are additional reports of lateralized stimulation-induced freezing and hesitant gait following bilateral STN placement. Specifically, FoG increased significantly with either left or bilateral stimulation, while FoG decreased with right STN stimulation alone or when bilateral stimulation was turned off. It was revealed that anteromedial placement of the left electrode compared to the planned target, possibly affecting the projections from the globus pallidus to PPN increasing FoG (19). High-frequency stimulation in the STN area (i.e., a region encompassing the STN, the ZI, and the fields of Forel) can selectively worsen on-med akinesia and gait while simultaneously improving rigidity and tremor (20). For this patient, no differences were seen with individual left or right DBS programming changes. Based on postoperative MRI, the left lead was slightly anterior compared to the right lead (Figure 1A). This supports prior reports suggesting that stimulation-induced akinesia and worsening gait were associated with stimulation in the most dorsal and anterior STN (21).

Based on our fiber tracking assessment, decreasing connectivity of the left VTA with the SMA, PFC, and cerebellum corresponded to improvement in the “on” FoG. We hypothesize that the PFC and cerebellum are the key areas that are involved in triggering “on” FoG in the setting of levodopa intake and high-frequency STN neurostimulation. The PFC is highly sensitive to a high dopaminergic environment given a large number of dopamine receptors that are present (22). Excessive dopaminergic stimulation can also cause deleterious effects on PFC function because of its inverted-U-shaped action (22, 23) as the threshold for FoG is not a linear phenomenon like dyskinesia (24, 25). Furthermore, long-term supratherapeutic levodopa is capable of influencing the frontal area function. It has been postulated that negative effects of levodopa on frontal executive functions can contribute to the occurrence of levodopa-induced FoG, although relatively uncommon, has been well-documented in selected cases (26, 27). Thus, it may be helpful to reduce the effects of dopamine on the PFC as seen in our patient by smoothing out the drug regimen, reducing dose-related peaks, and potentially reducing the overall daily levodopa dose.

Another study revealed that patients with “on” FoG had less activation of the posterior parietal cortex and less deactivation of the dorsolateral PFC and thalamus, and increase activation in the supplementary motor area (28). The supplementary motor area has direct projections to the STN responsible for strong inhibition of a planned action (29, 30) and is strongly activated during real FoG episodes (31). Several studies have shown that STN-DBS can improve the levodopa-responsive FoG, but has limited effect on “on” FoG (25). Moreover, degeneration of the prefrontal and frontal cortex may explain the levodopa-resistance symptoms and worsening of gait and FoG caused by stimulation of descending connections to the PPN.

Our study provides additional information by suggesting an important role of the cerebellum. Recent work has emphasized the tight interplay between the cerebellum and the basal ganglia (32, 33). Cerebellar activity is increased in PD patients, and hyperactivation in the cerebellum may be a compensatory mechanism for defective basal ganglia input (34–36). Based on these findings, medial cerebellum and its connections might be critical for balance and gait control in PD (37, 38). Experimental evidence point to the importance of the cerebellum locomotion region (CLR) in gait control, a region which exists in the midline of the cerebellum (39). Functional reorganization within the locomotor network which contains the SMA, the mesencephalic and the cerebellar locomotor regions has also been described in PD-FoG (40). More recently, Fasano et al. mapped lesions causing FoG using a common brain atlas, they observed that lesions to multiple different brain areas causing FoG were part of a common functional network connected to a focal area in the dorsal medial cerebellum, corresponding to the CLR (41). They suggested that lesion-induced FoG which is likely the “resistant” or “unresponsive” subtype (42), may be more likely to occur when lesions disrupt the medial cerebellum connections. In addition to the CLR, the mesencephalic locomotor region (MLR) is clearly important for gait, and its PPN component plays a part role in gait and postural control in patients with PD (23). Although we were unable to assess other subcortical connectivity in our case, future studies are necessary to delineate the relationship between “on” FoG and brain connectivity.

## Conclusions

According to our findings, we conclude that the interaction of impulsive dopaminergic stimulation and high-frequency STN stimulation could induce “on” FoG. The PFC and cerebellum might be the key areas that the combination of medication and STN-DBS effect to trigger this motor phenomenon. Nevertheless, few cases have been reported describing this association and assessing the mechanism of “on” FoG and further studies are required to confirm potential brain network connectivity patterns.

## Data Availability

The datasets generated for this study are available on request to the corresponding author.

## Ethics Statement

All procedures were approved by the Ethics Committee of Xuanwu Hospital of Capital Medical University. The subject gave written informed consent in accordance with the Declaration of Helsinki. And she gave written informed consent for publication of this report as well.

## Author Contributions

SM and AR-Z were the major contributors in writing the manuscript. PC, YZ, JL, and SM contributed to the diagnosis and treatment of the patient. EM contributed to the image analysis. LA, WH, AR-Z, and PC contributed to checking the manuscript. All authors read and approved the final manuscript.

### Conflict of Interest Statement

The authors declare that the research was conducted in the absence of any commercial or financial relationships that could be construed as a potential conflict of interest.

## Abbreviations

FoG: freezing of gait
VTA: volumes of tissue activated
STN-DBS: subthalamic nucleus deep brain stimulation
PPN: pedunculopontine nucleus
MDS-UPDRS: Movement Disorder Society Sponsored Revision of the Unified Parkinson's Disease Rating Scale.
